# State-of-the-Art and Prospective of Nanotechnologies for Smart Reproductive Management of Farm Animals

**DOI:** 10.3390/ani10050840

**Published:** 2020-05-13

**Authors:** Nesrein M. Hashem, Antonio Gonzalez-Bulnes

**Affiliations:** 1 Department of Animal and Fish Production, Faculty of Agriculture (El-Shatby), Alexandria University, Alexandria 21545, Egypt; 2 Departamento de Reproducción Animal. INIA, Avda. Puerta de Hierro s/n., 28040 Madrid, Spain; bulnes@inia.es; 3 Facultad de Veterinaria, Universidad Complutense de Madrid, Ciudad Universitaria s/n., 28040 Madrid, Spain

**Keywords:** hormone, nanotechnology, pregnancy, reproduction, semen

## Abstract

**Simple Summary:**

The present review aims to introduce current knowledge and prospective applications of nanotechnology for improving some of major applied assisted reproductive techniques (ART) in farm animals. Throughout the review, the raised question will be how nanotechnology, as a new emerging biotechnology, can be used to address the concept of smart reproductive management. The hypothesis will be discussed in the light of the few published studies in this field. This is in terms of leading the way for reproductive biologists to develop and innovate ART using nanotechnology scientific bases to maximize reproductive performance of farm animals, which finally serves the increasing consumer demand for animal products.

**Abstract:**

Many biotechnological assisted reproductive techniques (ART) are currently used to control the reproductive processes of farm animals. Nowadays, smart ART that considers technique efficiency, animal welfare, cost efficiency and environmental health are developed. Recently, the nanotechnology revolution has pervaded all scientific fields including the reproduction of farm animals, facilitating certain improvements in this field. Nanotechnology could be used to improve and overcome many technical obstacles that face different ART. For example, semen purification and semen preservation processes have been developed using different nanomaterials and techniques, to obtain semen doses with high sperm quality. Additionally, nanodrugs delivery could be applied to fabricate several sex hormones (steroids or gonadotrophins) used in the manipulation of the reproductive cycle. Nanofabricated hormones have new specific biological properties, increasing their bioavailability. Applying nanodrugs delivery techniques allow a reduction in hormone dose and improves hormone kinetics in animal body, because of protection from natural biological barriers (e.g., enzymatic degradation). Additionally, biodegradable nanomaterials could be used to fabricate hormone-loaded devices that are made from non-degradable materials, such as silicon and polyvinyl chloride-based matrixes, which negatively impact environmental health. This review discusses the role of nanotechnology in developing some ART outcomes applied in the livestock sector, meeting the concept of smart production.

## 1. Introduction

The current review aims to afford and connect the current knowledge in two emerging fields, which may become essential for animal production in the next few years: smart reproductive management and nanotechnology.

The picture for animal production in these early years of the 21st century is far from the scenario previously settled up during the 20th century. Animal production in the 20th century, agriculture on the whole, was influenced by the need of providing food and clothes to a growing population after a period of wars in the developed countries and after substantial changes in demographics and lifestyle in the developing areas. Strategies for providing food and clothes were implemented at any cost and included intensification of the use of natural resources to the limit and the extensive use of chemical substances (pesticides, antimicrobials or hormones), which act as xenobiotics.

The needs of world population will continue growing during the present century. The Food and Agriculture Organization (FAO) estimates a population of about 8500 million in 2030 and around 9150 millions of people in 2050, which means that food production should be increased by 70% (http://www.fao.org/fileadmin/user_upload/esag/docs/AT2050_revision_summary.pdf). However, currently, we have learnt that natural resources are limited and that any action on the environment has long-lasting consequences. Hence, agriculture needs to adapt to a changing environment and to avoid further changes in such environment, by improving its efficiency and sustainability and reducing its impact [1]. Moreover, animal production is strongly confronted by active movements against the use of animals in food and clothes production, which stresses the need to reinforce ethical aspects.

The same applies, within animal production, for reproductive management. The optimization of productive periods of breeding animals is a mandatory aim, not only for economic, but also by environmental reasons. Economically, the equilibrium between food and management inputs and productive outputs is compromised in animals with delayed puberty or extended intervals between weaning and conception; this is important for the economic profit of companies but also for the economic sustainability of small producers. Environmentally, these animals are only using natural resources to produce greenhouse gases and environmental waste without any benefit. Hence, the optimization of reproductive management is a must.

Reproductive management, and specifically the use of assisted reproductive technologies (ART), is a fast-growing field, which is essential for the manipulation of reproduction in the livestock sector. The main aim is to develop and implement relevant tools for improving fertility of farm animals with easily applied, affordable and effective techniques. Currently, such philosophy has to take into account the concept of smart production, which incorporates not only concepts as technique-efficiency and cost-efficiency, but also animal welfare, human health and environmental safety [2]. Therefore, great efforts are made by scientists to achieve the aims of smart production, particularly in the field of animal reproduction and specifically in the management of the reproductive activity, artificial insemination (AI), multiple ovulation and embryo transfer (MOET) and the management of pregnancy. These areas make necessary the administration of hormones, growth-factors and other substances and the manipulation of living cells like spermatozoa, oocytes and embryos. The efficiency of these processes is compromised by technical and economic issues, but also by environmental and health concerns, and all these aspects may be improved by the use of nanotechnology.

**Figure 1:**
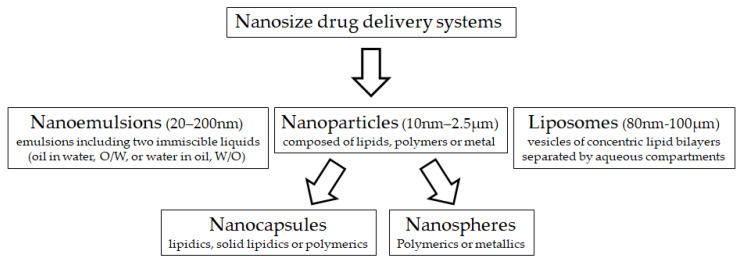
Main nano-sized material used for drug delivery systems.

Nanotechnology is an innovative discipline, combining the sciences of physics, chemistry, biology, mathematics as well as engineering and computer sciences. Nanotechnology has been used for the development of diagnostic and therapeutic agents in human medicine, but its application in animal medicine and production is still scarce. The concept of this technology is to transform chemical molecules into small-scale size particles (range 1–200 nm). Consequently, new physical and chemical properties are achieved, including greater cellular uptake, reactivity, surface area and charge, and binding properties which might have opened a new platform for biosciences innovation [3,4]. Nano-sized drug delivery systems (Figure 1) are being developed, both as therapeutic agents and as therapy delivery systems, because of the use of germicidal properties of metal and polymeric nanoparticles as antimicrobials, while natural and nanostructured materials can entrap and protect compounds for delivery [5]. Many nanomaterials may be useful to overcome some obstacles that compromise the efficiency of ART in animal production.

Specifically, for the male, at post-collection semen handling for AI, specific nanomaterials are being designed to obtain semen doses with high sperm quality, by optimizing both semen purification (removing of defective sperm cells) and semen preservation (cooling or freezing) processes. For example, nano-magnetic pods of iron oxide (Fe_3_O_4_ NPs), characterized by their bio-compatibility and bio-function [6], have been used to purify both fresh semen ejaculates and frozen-thawed semen previously to be applied in the field scale AI or in vitro fertilization (IVF), respectively. The first studies indicate the ability of producing semen doses with high recovered purified sperm cells in boars [7] and increased conception rates following AI in cattle [8]. Nanoparticles of compounds with high antioxidant activity such as cerium oxide [9], selenium [10] and zinc [11] can be also used as semen extender additives, to protect frozen semen from the negative effects of reactive oxygen species during the cryopreservation process.

In the case of females, the management of the reproductive cycle for estrus synchronization, AI and MOET, relies on the use of exogenous hormones [12,13]. However, the effectiveness of hormone-based protocols is limited by technical and economic issues (biological activity of the hormone and the cost of protocol). Concomitantly, there are concerns regarding the presence of hormonal residues in animal products (milk or meat) and the release of hormonal wastes and discharges to the environment (non-degradable hormone carriers), which impacts public health and environmental safety. The use of nano-hormone delivery system (HDS) may allow better bioavailability with lower doses [4], improved animal welfare [14] and the decreased risk of environmental pollution by hormonal residues [4,15]. Furthermore, the use of nanotechnology for the management of pregnancy complications constitutes a new and promising field; most of the studies are focused on the use of nanoparticles for a targeted delivery of therapeutics to the placenta. However, the inherent risk of fetal damage makes the development of extensive research in the area prior to its practical application necessary. Finally, the ecological footprint of nanotechnology-based protocols should be well investigated prior to their application in field scale, insuring the safety of used materials and their residues for environment, human and animals.

Hence, the present review assesses the efficacy and feasibility, for the livestock sector, of nanotechniques that have been developed to improve some ART outcomes and points out possible prospective applications of nanotechnologies focused on smart livestock production.

## 2. Nanotechnologies for Post-Collection Semen Handling

### 2.1. Semen Preservation

One of the most applied ART in male reproduction is semen preservation, either by cooling or cryopreservation. However, the efficiency of both techniques is still challenged, because of their negative impact on sperm quality. Both techniques are known to increase reactive oxygen species (ROS) and therefore to evoke oxidative stress in spermatozoa, impairing integrity of spermatozoon membranes, mitochondrial membranes and nuclear DNA content, and thus leading to a significant reduction in its aptitude to fertilize the oocyte [16].

During cooling/freezing processes, spermatozoa are kept in synthetic extenders which are created to provide nutrients and also to protect against cold/freezing shock and microbial attack. Thus, there have been intensive attempts to ameliorate negative effects of cooling/freezing preservation techniques on spermatozoa structure and function, by manipulating the chemical composition of semen extenders. In this respect, nanotechnology has been employed to modify semen extender properties, improving the uptake of nutrients and/or supplemented active components. 

One interesting study in this field was carried out by Murawski et al. [17], who declusterized water by using a cold plasma reactor to produce nanowater (NW), to be used instead of deionized water (DW) as an extender media. Compared to DW, NW has many new properties; mainly, high diffusivity, low viscosity, very low density, low dielectric constant and zero coefficient of thermal expansion at freezing. Thus, it can dissolve even non-polar compounds (lipids) into solution, being more efficient as a carrier of nutrients and inorganic compounds than other normal water. In addition, it possesses antimicrobial properties. In this study, the authors stated that NW-containing media substantially improved the fertilizing ability of frozen-thawed ram semen and lamb productivity of inseminated ewes.

Enrichment of the semen extender with antioxidant agents, such as antioxidant minerals, has been reported to improve semen quality properties of cooling or post-thawing sperm cells, particularly if they are in nanoforms. It is currently known that the supplementation of semen extenders with cerium oxide nanoparticles (CeO_2_ NPs, an oxygen storing molecule) during semen storage at 4 °C for 96 h improves sperm motility characteristics after 48 h, and even up to 96 h of incubation, and protects the integrity of plasma membranes and DNA of spermatozoa [18]. Nanoparticles of selenium (Se NPs) have also been used in several studies as a ROS scavenger to protect against oxidative damage in sperm cells. Khalil and coworkers [10] found that the enrichment of semen extenders with Se NPs at a concentration of 1.0 mg/mL improved post-thawing sperm quality in Holstein bulls and, consequently, in vivo fertility rate by reducing apoptosis, lipid peroxidation and sperm damage occurring by cryopreservation. Similarly, the supplementation of bull semen extender with zinc nano-complex (Zn NPs) during cryopreservation has been found to decrease lipid peroxidation and improve the mitochondrial activity and functionality of sperm plasma membrane in a dose-dependent manner without any deleterious effect on motility parameters [11]. 

The semen cooling/freezing process may also harmfully affect the spermatozoa cytoplasmic membrane by re-localization of phospholipids into a different arrangement. In this respect, phospholipids-based NPs have been found to improve spermatozoa membrane stability by compensating the free fatty-acids and phospholipids removed from spermatozoa membrane during cryopreservation. Nadri and coworkers [19] reported that the use of a nano-lecithin-based extender (2% lecithin) for the dilution of goat semen improves sperm cryosurvival (in terms of lower apoptosis and higher motility, viability and sperm membrane functionality), when compared to lecithin-based and egg yolk-based extenders.

A summary of some studies connecting nanotechnology and semen preservation is shown in Table 1.

### 2.2. Semen Purification

Semen purification techniques represent a valid tool for the selection of sub-populations of best spermatozoa (in terms of motility and morphology) and the removal of defective cells, contaminations and debris within an ejaculate. These techniques may be especially helpful in recovering spermatozoa from sub-optimal ejaculates from animals of high genetic values but, moreover, removal of dead spermatozoa is highly beneficial, because of the negative effects on motility and membrane integrity of live spermatozoa [22]. The main techniques for semen purification in farm animals are density gradient centrifugation [23], swim-up assay [24], column purification [25] and single layer centrifugation [26]. Although these techniques significantly improve the motility and functional parameters of sperm cells in semen doses, especially in post-thawing semen, their efficiency is still limited by variable recovery rates (leading to low sperm concentration in semen doses), long run time and increased costs and labor [27].

A novel approach for semen purification that relies on the detection of specific biomarkers raised by defective spermatozoa is developed [28]. Using fundamentals of cell-specific targeting nanotechnologies, defective spermatozoa can be precisely eluted from a semen pool. For this purpose, iron oxide nanoparticles (Fe_3_O_4_ NPs) that are characterized by their small size (10–100 nm), great superparamagnetism and surface area and bio-compatibility and bio-functionalization properties have been employed as an easy separation method. These NPs can be functionalized with specific probes and directed to defective spermatozoa using an external magnetic field, allowing the separation of defective spermatozoa [6]. The identified biomarkers expressed by defective spermatozoa and the corresponding functionalized Fe_3_O^4^ NPs probes used for semen purification are shown in Table 1.

Studies in the purification of semen of farm animals have supported the efficiency of this technique; Fe3O4 NPs functionalized with probes of peanut agglutinin lectin (PNA), *Pisum sativum* agglutinin lectin (PSA), annexin V and anti-ubiquitin antibodies have been successfully used in bulls for removing defective spermatozoa [29]. In context, Odhiambo and coworkers [8] have developed two types of functionalized Fe_3_O_4_ NPs to purify frozen-thawed bull spermatozoa: a particle coated with antibody against ubiquitin, which is present on the surface of defective spermatozoa, and a particle coated with the PNA, which binds to glycans exposed by acrosomal damage. The results of AI in 798 cows in a field trial showed that the conception rate obtained using 10 × 10^6^ PNA-nano-purified sperm doses (64.5%) was similar to the rate obtained using 20 × 10^6^ non-purified full sperm doses (63.3%) and significantly higher than using a 10 × 10^6^ non-purified sperm dose (53.7%). Similarly, Fe_3_O_4_ NPs coated with PNA/PSA lectins were used for the nanopurification of boar semen and resulted in semen doses with high sperm motility and viability [7]. 

In summary, previous studies have shown that sperm purification using nano-magnetic based techniques may overcome the inconveniences of other semen purification techniques such as extensive semen manipulation, labor and additional costs and run time (> 1 h to prepare a purified semen sample), without needing expensive equipment [27]. These promising results suggest that nanotechnologies could be easily included in the routine procedures of selection of high quality sperm population to enhance the fertility of farm animals.

## 3. Nanotechnologies for Management of Cycle and Implementation of ART in Females

In this section, the advantages of nano-drug delivery technologies for smart management of the reproductive cycle in females will be discussed, with particular focus on the mechanisms involved. In conventional drug delivery systems, the bioavailability of the drug relies on several factors, such as the permeability throughout the epithelial and endothelial cells, the solubility, the passage through blood barriers, the clearance rate by liver and kidney and the resistance against systemic and circulating degradation enzymes. Thus, smart nanostructured delivery systems, particularly those depending on the polymerization of bioactive molecules using biodegradable and biocompatible polymers, are engineered with different physicochemical properties acting via different mechanisms, to efficiently deliver drugs from the site of administration to the sites of action, overcoming the unfavorable drug properties and the biological barriers [30]. 

One of the mechanisms by which NP-based drug delivery systems can protect drugs from enzymatic degradation and rapid clearance depends on the ability of the nanoformula to absorb and/or encapsulate the drugs. The other mechanisms by which NP-based drug delivery systems can improve drug pharmacokinetic and pharmacodynamic properties are related to the physicochemical properties of the nanoformula such as shape, particle size, surface charge (zeta potential) and particles polydispersity (PdI). Particle size can play significant roles in the determination of cellular uptake and degradation and elimination processes of the drug. Small sized-Nps can escape from reticuloendothelial and mononuclear phagocytic systems, leading to an increase in total blood circulation time and bioavailability. Additionally, surface charge characteristics can affect the degree of NPs uptake according to the charge of tissue cells. Many cellular membranes are negatively charged; thus, cationic NPs facilitate membrane attraction and adhesion, which create favorable properties for cellular uptake, via endocytosis or other mechanisms [31].

Based on the previously mentioned scientific facts, many drugs used for managing reproductive processes in females were developed to have new properties meeting the smart reproductive management aims.

### 3.1. Nano-Hormone Delivery Systems and Cycle Management

Procedures for implementing ART in farm animals are based on the administration of exogenous reproductive hormones (mainly gonadotrophins, steroids and prostaglandins) for controlling the reproductive cycle, improving reproductive efficiency and treating some reproductive disorders [12,32,33]. However, the effectiveness of hormonal treatments for controlling farm animals’ reproduction is limited by several factors [2,14,34]: 1. the biological activity of the hormone (which depends on its bioavailability, kinetics and dose) 2. the animal response to the hormonal treatment after repeated treatments (some hormones evoke the formation of antibodies); 3. the cost of the applied protocol; 4. animal health and welfare; 5. environmental-related issues (release of xenobiotics and also waste to the environment in the case of undegradable hormone carriers); and 6. consumers’ health (raising concern about residual effect in tissues and thus the possibility of consumption of animal products with hormonal residues).

A summary of studies connecting the nanotechnology and management of female reproduction is shown in Table 2. The use of nano-drug, or nano-hormone delivery systems (NHDS) may be useful for alleviating the incidence of these factors. Firstly, enhancing the biological efficiency of the hormonal treatment; conjugation of the hormone with a suitable nano-particle is hypothesized to increase its half-life, improve its passage across epithelial or endothelial barriers into blood or lymph circulation and sustain its delivery to the target sites, improving their uptake by cells [35]; hence, it is possible to use lower doses. As an example, gonadotrophin releasing hormone (GnRH) is included in some prostaglandin-based protocols, since it induces ovulation and has luteotrophic effects favoring progesterone secretion and the maintenance of pregnancy [36]. However, GnRH has a short half-life time in blood circulation, which diminishes both its biological activity and sustained action. Hashem and Sallam [4] found that the administration of chitosan- sodium tripolyphosphate (TPP)-conjugated GnRH nanoparticles in goats allowed 75% reduction in the GnRH dose, without affecting fertility and prolificacy, which supports that the nanoformula increases the bioavailability of the hormone. In this study, the main proposed mechanism involved in the reduction of the GnRH dose was the ability of nanoformula to increase the bioavailability of the GnRH to the brain (pituitary, target organ) as the size, PdI and zeta potential of fabricated chitosan-TPP-conjugated gonadorelin NPs were 93.91nm, 0.302 and 11.6 mV, respectively, with 91.2% entrapment efficiency for GnRH. NPs of a size ranging from 50 to 200 nm, a PdI less than or equal to 0.3 and a low positive charge (up to 15 mV) are efficient for drug delivery to the brain [37].

In addition to allowing the use of a decreased dose, NHDS may also be used to change the route of administration [36] and therefore increase animal welfare and decrease the risk of exposition to different hormones by workers and technicians [38]. Specifically, protocols for AI in rabbits usually include GnRH-supplemented extenders to induce ovulation via semen dose. This is certainly a good example of a welfare-orientated method, being a non-invasive route which diminishes animal distress and, labor amount and working time when compared to conventional treatments via intramuscular doses. However, the biological barriers for mucosal permeation and the proteolytic activity of seminal plasma and vaginal fluids limit this technique and, to achieve fertility results similar to the conventional intramuscular protocol, the hormone concentration administered intravaginally should be about ten-folds higher, which constitutes a potential health risk for workers. As an attempt to alleviate these risks, chitosan–dextran sulphate GnRH (buserelin acetate) NPs have been fabricated and supplemented to semen extender of rabbits, which allows a 50% of reduction in the dose without affecting fertility [14]. Similar results were obtained by Hassanein et al. [39] when chitosan-TPP GnRH NPs were used to induce ovulation by applying the chitosan-TPP GnRH NPs intramuscularly or intravaginally during AI process. These results were attributed to the protective role of chitosan and dextran sulfate NPs for GnRH against the negative action of vaginal proteolytic enzymes, and the improved mucoadhesive properties of the nanoformula [14]. Cationic charged polymers such as chitosan yield an attractive force with the anionic polyelectrolyte properties of vaginal mucus, resulting in enhanced muco-adhesion and the retention of NPs within the mucus layer, allowing both sustained release and higher cellular uptake by vaginal mucosa [31].

Finally, conventional hormonal treatments are also upsetting environmental issues, due to the release of hormonal residues and carrier materials into the environment. The most evident example is the use of progesterone-impregnated intravaginal devices (e.g.,: CIDR, PRID and Cue-Mate) which were developed for cattle, and afterwards for small ruminants, since the 1970s and which are currently available worldwide [40,41]. The main matrix of these devices is composed of silicon polymers, which need to be loaded with high progesterone concentrations to release enough hormones to the vaginal mucosa. Progesterone levels into the device remain high after discard and currently, inserts based on polyethylene vinyl acetate (EVA) copolymers polyisoprene and polymethyl-methacrylate are being developed for reducing progesterone charge, thus diminishing the costs and emissions of hormones to the environment [2,42]. However, similarly to silicone-based devices, these materials are not biodegradable. In view of these circumstances, biodegradable and biocompatible polymeric materials, such as chitosan and poly (lactic acid) (PLA), poly (glycolic acid) (PGA) and PLGA polymers are being tested. 

In view of these circumstances, Helbling and coworkers [43] developed a complex of chitosan-tpp-Tween80 as a biodegradable carrier for progesterone using the spray-drying technique. The particles obtained by this method were at micro size (ranged between 1 and 7 μm) and 69%–75% encapsulation efficiency; however, the authors supported its efficacy as a biodegradable delivery system for progesterone in cattle. Oliveira and coworkers [15] developed and tested biodegradable and biocompatible nanomaterials (specifically nanofibrous mats of polylactic acid; PLA) loaded with progesterone by solution blow spinning technique, with promising results for controlled progesterone delivery. Similarly, Fogolari and coworkers [42] used a method of miniemulsion polymerization for producing two forms of progesterone-conjugated nanocapsules: nanospheres (NS) and nanocapsules (NC), using polymethyl-methacrylate, a biocompatible polymer, instead of silicone-based release devices. The encapsulation efficiencies of NS and NC were greater than 69% and 90%, with average sizes of 150–200 nm and 240–300 nm, respectively, which supports the usefulness of this method for progesterone binding.

However, the few studies performed in this respect have shown promising results regarding the replacement of non-biodegradable progesterone carriers with biodegradable ones using advances of NHDS; the application of such technology on farm scale needs more efforts to be justified [43]. This is simply because the use of vaginal drug nanocarriers has to consider many biological mechanisms, including the protection of labile molecules from adverse environmental agents, controlled release, the modulation of adhesion to mucus, mucosal tissue penetration, specific targeting, and intracellular delivery.

### 3.2. Nano-Drug Delivery Systems and Management of Pregnancy 

Pregnancy and delivery are pivotal facts for the reproductive management and productivity of farm animals. One of the main complications of pregnancy in most farm animals, as in other many species including humans, is the occurrence of intrauterine growth restriction (IUGR) [45,46,47]. IUGR is the failure of a fetus to reach its full genetic growth potential [48]) and its appearance may be related to genetic traits, infections or, more frequently, to an intrauterine environment inadequate for the development of the fetus. The main factor affecting the intrauterine environment is the availability of nutrients and oxygen required by the fetus for its development, so maternal undernutrition, heat stress and/or hypoxia are the main causes of IUGR. The placenta is the organ driving the transfer of nutrients and oxygen from the mother and the fetus, so alterations in placental development (by the crowding of uterine space in case of twins in ruminants or hyperprolificacy in pigs and rabbits) and placental efficiency (by inadequate function) are also main causes for IUGR. 

The main therapies for IUGR, in the case of farm animals, are based on maternal nutritional supplementation with amino acids favoring placental and fetal neoangiogenesis and tissue development, with vitamins favoring protein synthesis and antioxidant capacity and with other antioxidant agents like polyphenols [31]. In humans, pharmacological treatments including the use of aspirin, sidenafil citrate or metformin are also applied. However, all these therapies are based on actions at the systemic level, which makes the administration of these substances at high doses to the mother to allow their availability at the fetoplacental level necessary, with the obvious risk to mother and fetus. Future prospects are based on focused treatments, either directly at the fetus, or by using infusion of umbilical cord, amniotic sac or placenta, with lower risk. 

In this field, the infusion of the therapies throughout the umbilical cord, the amniotic sac or the placenta may be clearly optimized by the used nanoparticles. Nanoparticles can be directly used as a tool for the targeted delivery of therapeutics to the placenta. In this sense, surface-functionalized nanoparticles focused on receptors exclusively expressed at the placenta are giving promising results for their future use in the targeted therapy of pregnancy complications. In this sense, we have to highlight the works using plCSA binding peptide (plCSA-BP) nanoparticles [49], which target the placenta via placental chondroitin sulfate A (plCSA). These strategies would enable one not only to direct the drug to the fetus, but also to increase the effectiveness and specificity of the carried compound and to decrease collateral effects [50]. Prospective studies for possible treatments with nanoparticles have been based mainly on the administration of growth factors and nitric oxide donors [51].

On the other hand, pregnancy complications may not directly affect the fetus but indirectly, through negative effects on the the maternal side, the uterus and maternal placenta. In this case, the objective would be to treat the mother without affecting the fetus. A classic example is preterm labor, which needs to be treated with tocolytic drugs like indomethacin which freely crosses the placenta and seriously affects the fetus (narrowing the *ductus arteriosus* and causing hydramnios, enterocolitis and hemorrhagiae). The objective of nanotechnology, in this case, would be to use molecules of a larger size that do not cross the placenta and act focally on the uterus [52]. A good example is the work of Paul et al. [53], by developing immunoliposomes (liposomes that use antibodies for targeting a specific organ or condition) loaded with tocolytic drugs and conjugated to oxytocin receptor antibody, for targeting the oxytocin receptor on the pregnant uterus. 

However, fetal treatment with nanoparticles is at its very early beginning and, having in mind possible harmful effects, extensive preclinical studies should be undertaken prior to its practical application.

## 4. Concluding Remarks

The applications of nanotechnology in the field of animal reproductive science could contribute to improve different reproductive assisted techniques to be more efficient, practicable, cost-efficient and safe for environmental health and animal welfare, dealing with the smart reproductive management aspects. However, there is still the need for extensive studies on limitations and prospective applications prior to its practical application. There are still important questions, regardless of the progression of nanotechnology-based ART discussed in this review, within this field, that remain to be scientifically answered. Specifically, we must clarify how much nanotechnology-based ART can meet the concept of smart ART. For example, more information is required regarding the fate, toxicity and kinetics of different types (inorganic and organic-based NPs) of NPs, in both biological systems and environments [54]. This may require multidisciplinary cooperation in order to assess the release, transportation, transformation, accumulation, toxicity, and uptake of engineered NPs from the biological systems (animal) into the environment. Until finding answers for such questions, studies that have been carried out in the field of nanotechnology-based ART expected the practical application of some of these technologies in the near future including semen preservation, sperm purification and NHDS [12,14,16], following the evaluation of their efficiency and cost in a large field scale.

## Figures and Tables

**Table 1:** Summary of previous research connecting nanotechnology and semen preservation and purification.

Animal Species	Nanoparticle (NPs), Level ^1^	Assisted Reproductive Technologies	Smart Target	Result ^1^	Reference
Pigs	Fe_3_O_4_ NPs coated with lectins or annexin V 0, 87.5, and 175 μg/mL	Purification of fresh or extended semen	Rapid, efficient and safe purification	Fe_3_O_4_ NPs coated with lectins or annexin V at 87.5 μg /mL: Improved sperm motility and viability with no negative effects on sperm acrosome, plasma membrane, and mitochondrial membrane integrity No negative effects on fertility of inseminated gilts and of offspring performance	[20]
Bulls	Fe_3_O_4_ NPs coated with avidin+ DNA aptamers	Purification of unsorted and sex-sorted sperm cells	Rapid, efficient, safe and inexpensive semen purification technique for large scale application	Improved unsorted and sex-sorted sperm quality No negative effects on in vitro embryo development	[21]
	Fe_3_O_4_ NPs coated with lectin or ubiquitin antibodies 0.1 mg/ml	Purification of cryopreserved semen for IVF Purification of fresh semen for AI field trail		Improved oocyte IVF rate Fe_3_O_4_ NPs coated with lectin reduced semen dose required for AI	[8]
Rams	Cerium oxide (CeO_2_) NPs 0, 44 and 220µg/mL	Semen cooling at 4 °C	Sufficient post-storage semen quality	Increasing levels of CeO_2_ NPs: Improved kinematic and morphologic variable No genotoxic effects of CeO_2_ NPs	[9,16,18]
Rams	Nanowater (NW) NW as extender media	Semen cryopreservation	Minimizing cryopreservation impacts on post-thawing sperm quality	Decreased aspartate aminotransferase and alkaline phosphatase concentrations Improved sperm fertilizing ability Increased conception and lambing rates	[17]
Goat bucks	Nano- lecithin 1, 2, 3 and 4% of semen extender			Nano-lecithin at 2%: Improved post-thawing sperm quality (higher motility, viability and HOST, and lower apoptosis). No effects on embryo cleavage or blastocyst ratios in IVF	[19]
Bulls	Selenium NPs 0, 0.5, 1.0 and 1.5 µg/mL extender			Selenium NPs at 1.0 µg/mL: Improved post-thawing kinematic and morphologic sperm quality Decreased apoptotic and necrotic sperm cells Improved seminal plasma antioxidant status Increasing in vivo fertility rate	[10]
	Zn- nano- complex 0, 10^−6^, 10^−5^, 10^−4^, 10^−3^, 10^-2^ molar/ml			Increasing levels of Zn- nano- complex: Improved plasma membrane functionality and mitochondrial activity No deleterious effect on motility parameters	[11]

^1^ AI = artificial insemination; IVF = in vitro fertilization^.^

**Table 2:** Summary of previous research connecting nanotechnology and female reproduction.

Animal Species	Nanoparticle (NPs), Dose, Route of Administration ^1^	ART	Smart Target ^2^	Result	Reference
Goats	GnRH(gonadorelin)-chitosan-Tpp NPs 0 or 50 µg bare GnRH/doe i.m. versus 12.5 µg conjugated GnRH/doe i.m.	Cycle control/ovulation induction	Reduce hormone dose Improving ovulation rate and luteal fuction	Improved ovulation rate, luteal function and prolificacy 75% reduction in hormone dose	[4]
Dairy cow	hCG(Choluron)-chitosan-Tpp NPs 1000 IU bare hCG/cow, i.m. versus 1000 IU conjugated hCG/cow, i.m.		Change route of administration (nasal spray)	Compared to i.m. treatment, no effects on time of ovulation, follicle size, corpus luteum size and onset of estrus	[44]
Rabbits	GnRH (buserelin acetate)-chitosan-Tpp NPs 0.8 bare GnRH/doe i.m. versus 0.2 or 0.4 µg conjugated GnRH/doe i.m. and 4 or 8 µg conjugated GnRH/doe i.va.	AI/Ovulation induction	Change route of administration (vaginal adsorption) Reduce hormone dose Reduce AI steps Animal welfare	Ovulation was induced successfully except for 2 µg conjugated GnRH/doe i.va. 75% reduction in i.m. hormone dose 50% reduction in i.va. hormone dose	[37]
	GnRH (buserelin acetate) -chitosan- dextran sulfate NPs 4 or 5 µg bare GnRH /doe i.va. versus 4 or 5 µg conjugated GnRH /doe i.va.			Compared to 4 µg bare GnRH/doe, other treatments increased fertility Reduction in the GnRH conventional dose via vaginal administration	[14]
Livestock animals	Poly(lactic acid)-progesterone nanofibers	Cycle control	Elimination use of non-biodegradable devices and environmental health Animal welfare	Eligibility of nanofibers for controlled progesterone delivery	[15]

^1^ i.va. = intravaginal, i.m. = intramuscular. ^2^ AI= artificial insemination.

## References

[1] Martin G.B. (2014). An Australasian perspective on the role of reproductive technologies in world food production. Adv. Exp. Med. Biol..

[2] De Graaff W., Grimard B. (2018). Progesterone-releasing devices for cattle estrus induction and synchronization: Device optimization to anticipate shorter treatment durations and new device developments. Theriogenology.

[3] Feugang J.M., Rhoads C.E., Mustapha P.O., Tardif S., Parrish J.J., Willard S.T., Ryan P.L. (2019). Treatment of boar sperm with nanoparticles for improved fertility. Theriogenology.

[4] Hashem N.M., Sallam S.M. (2020). Reproductive performance of goats treated with free gonadorelin or nanoconjugated gonadorelin at estrus. Domest. Anim. Endocrinol..

[5] Hill E.K., Li J. (2017). Current and future prospects for nanotechnology in animal production. J. Anim. Sci. Biotechnol..

[6] Huang S.H., Juang R.S. (2011). Biochemical and biomedical applications of multifunctional magnetic nanoparticles: A review. J. Nanoparticle Res..

[7] Feugang J., Liao S., Crenshaw M., Clemente H., Willard S., Ryan P. (2015). Lectin-functionalized magnetic iron oxide nanoparticles for reproductive improvement. J. FIV Reprod. Med. Genet..

[8] Odhiambo J.F., DeJarnette J.M., Geary T.W., Kennedy C.E., Suarez S.S., Sutovsky M., Sutovsky P. (2014). Increased conception rates in beef cattle inseminated with nanopurified bull semen. Biol. Reprod..

[9] Falchi L., Bogliolo L., Galleri G., Ariu F., Zedda M.T., Pinna A., Malfatti L., Innocenzi P., Ledda S. (2016). Cerium dioxide nanoparticles did not alter the functional and morphologic characteristics of ram sperm during short-term exposure. Theriogenology.

[10] Khalil W.A., El-Harairy M.A., Zeidan A.E., Hassan M.A. (2019). Impact of selenium nano-particles in semen extender on bull sperm quality after cryopreservation. Theriogenology.

[11] Jahanbin R., Yazdanshenas P., Amin A.M., Mohammadi S.A., Varnaseri H., Chamani M., Nazaran M.H., Bakhtiyarizadeh M.R. (2016). Effect of zinc nano-complex on bull semen quality after freeze-thawing process. J. Anim. Prod..

[12] Hashem N.M., Aboul-Ezz Z.R. (2018). Effects of a single administration of different gonadotropins on day 7 post-insemination on pregnancy outcomes of rabbit does. Theriogenology.

[13] Hashem N.M., El-Azrak K.M., Sallam S.M. (2016). Hormonal concentrations and reproductive performance of Holstein heifers fed *Trifolium alexandrinum* as a phytoestrogenic roughage. Anim. Reprod. Sci..

[14] Casares-Crespo L., Fernández-Serrano P., Viudes-de-Castro M.P. (2018). Protection of GnRH analogue by chitosan-dextran sulfate nanoparticles for intravaginal application in rabbit artificial insemination. Theriogenology.

[15] Oliveira J.E., Medeiros E.S., Cardozo L., Voll F., Madureira E.H., Mattoso L.H., Assis O.B. (2013). Development of poly (lactic acid) nanostructured membranes for the controlled delivery of progesterone to livestock animals. Mater. Sci. Eng. C.

[16] Falchi L., Khalil W.A., Hassan M., Marei W.F. (2018). Perspectives of nanotechnology in male fertility and sperm function. Inter. J. Vet. Sci. Med..

[17] Murawski M., Schwarz T., Grygier J., Patkowski K., Oszczęda Z., Jelkin I., Kosiek A., Gruszecki T.M., Szymanowska A., Skrzypek T. (2015). The utility of nanowater for ram semen cryopreservation. Exp. Biol. Med..

[18] Falchi L., Galleri G., Dore G.M., Zedda M.T., Pau S., Bogliolo L., Ariu F., Pinna A., Nieddu S., Innocenzi P. (2018). Effect of exposure to CeO_2_ nanoparticles on ram spermatozoa during storage at 4 °C for 96 h. Reprod. Biol. Endocrinol..

[19] Nadri T., Towhidi A., Zeinoaldini S., Martínez-Pastor F., Mousavi M., Noei R., Tar M., Sangcheshmeh A.M. (2019). Lecithin nanoparticles enhance the cryosurvival of caprine sperm. Theriogenology.

[20] Durfey C.L., Swistek S.E., Liao S.F., Crenshaw M.A., Clemente H.J., Thirumalai R.V., Steadman C.S., Ryan P.L., Willard S.T., Feugang J.M. (2019). Nanotechnology-based approach for safer enrichment of semen with best spermatozoa. J. Anim. Sci. Biotechnol..

[21] Farini V.L., Camaño C.V., Ybarra G., Viale D.L., Vichera G., Yakisich J.S., Radrizzani M. (2016). Improvement of bovine semen quality by removal of membrane-damaged sperm cells with DNA aptamers and magnetic nanoparticles. J. Biotechnol..

[22] Brinsko S.P., Blanchard T.L., Rigby S.L., Love C.C., Varner D.D. (2003). Effects of dead spermatozoa on motion characteristics and membrane integrity of live spermatozoa in fresh and cooled-stored equine semen. Theriogenology.

[23] Sieme H., Martinsson G., Rauterberg H., Walter K., Aurich C., Petzoldt R., Klug E. (2003). Application of techniques for sperm selection in fresh and frozen-thawed stallion semen. Reprod. Domest. Anim..

[24] Arias M.E., Andara K., Briones E., Felmer R. (2017). Bovine sperm separation by Swim-up and density gradients (Percoll and BoviPure): Effect on sperm quality, function and gene expression. Reprod. Biol..

[25] Galarza D.A., Lopez-Sebastian A., Woelders H., Blesbois E., Santiago-Moreno J. (2018). Sephadex filtration as successful alternative to density-gradient centrifugation procedures for ram sperm selection with improved kinetics. Anim. Reprod. Sci..

[26] Nongbua T., Johannisson A., Edman A., Morrell J.M. (2017). Effects of single layer centrifugation (SLC) on bull spermatozoa prior to freezing on post-thaw semen characteristics. Reprod. Domest. Anim..

[27] Feugang J.M. (2017). Novel agents for sperm purification, sorting, and imaging. Mol. Reprod. Dev..

[28] Sutovsky P., Aarabi M., Miranda-Vizuete A., Oko R. (2015). Negative biomarker-based male fertility evaluation: Sperm phenotypes associated with molecular-level anomalies. Asian J. Androl..

[29] Faezah S.S., Zuraina F.M., Farah J.H., Khairul O., Hilwani N.I., Iswadi M.I., Fang C.N., Zawawi I., Abas O.M., Fatimah S.I. (2014). The effects of magnetic separation on cryopreserved bovine spermatozoa motility, viability and cryo-capacitation status. Zygote.

[30] Lombardo D., Kiselev M.A., Caccamo M.T. (2019). Smart nanoparticles for drug delivery application: Development of versatile nanocarrier platforms in biotechnology and nanomedicine. J. Nanomater..

[31] Cooper D.L., Conder C.M., Harirforoosh S. (2014). Nanoparticles in drug delivery: Mechanism of action, formulation and clinical application towards reduction in drug-associated nephrotoxicity. Expert Opin. Drug Deliv..

[32] Hashem N.M., El-Zarkouny S.Z., Taha T.A., Abo-Elezz Z.R. (2015). Oestrous response and characterization of the ovulatory wave following oestrous synchronization using PGF2α alone or combined with GnRH in ewes. Small Rumin. Res..

[33] Gonzalez-Bulnes A., Parraguez V.H., Berlinguer F., Barbero A., Garcia-Contreras C., Lopez-Tello J., Pesantez-Pacheco J.L., Martinez-Ros P. (2020). The impact of prenatal environment on postnatal life and performance: Future perspectives for prevention and treatment. Theriogenology.

[34] Rather M.A., Sharma R., Gupta S., Ferosekhan S., Ramya V.L., Jadhao S.B. (2013). Chitosan-nanoconjugated hormone nanoparticles for sustained surge of gonadotropins and enhanced reproductive output in female fish. PLoS ONE..

[35] Saha R., Bhat I.A., Charan R., Purayil S.B., Krishna G., Kumar A.P., Sharma R. (2018). Ameliorative effect of chitosan-conjugated 17α-methyltestosterone on testicular development in *Clarias batrachus*. Anim. Reprod. Sci..

[36] Hashem N.M., El-Azrak K.M., El-Din A.N., Taha T.A., Salem M.H. (2015). Effect of GnRH treatment on ovarian activity and reproductive performance of low-prolific Rahmani ewes. Theriogenology.

[37] Cláudia S., Ferreira C.B.R., Santos T., Ferreira L., Bernardino L. (2016). Nanoparticle-mediated brain drug delivery: Overcoming blood–brain barrier to treat neurodegenerative diseases. J. Control. Release.

[38] Tomoda K., Watanabe A., Suzuki K., Inagi T., Terada H., Makino K. (2012). Enhanced transdermal permeability of estradiol using combination of PLGA nanoparticles system and iontophoresis. Colloids Surf. B.

[39] Hassanein E.M., Hashem N.M., El-Azrak K.M., Hassan G.A., Salem M.H. Effect of GnRH–loaded chitosan-TPP nanoparticles on ovulation induction and embryo recovery rate using artificial insemination in rabbit does. Proceedings of the 10th International Poultry Conference.

[40] Roche J.F. (1978). Control of oestrus in cattle using progesterone coils. Anim. Reprod. Sci..

[41] Rathbone M.J., Burke C.R. (2013). Controlled Release Intravaginal Veterinary Drug Delivery. Long Acting Animal Health Drug Products.

[42] Fogolari O., Felippe A.C., Leimann F.V., Gonçalves O.H., Sayer C., Araújo P.H. (2017). Method validation for progesterone determination in poly (*Methyl methacrylate*) nanoparticles synthesized via miniemulsion polymerization. Int. J. Polym. Sci..

[43] Helbling I.M., Busatto C.A., Fioramonti S.A., Pesoa J.I., Santiago L., Estenoz D.A., Luna J.A. (2018). Preparation of TPP-crosslinked chitosan microparticles by spray drying for the controlled delivery of progesterone intended for estrus synchronization in cattle. Pharm. Res..

[44] Pamungkas F.A., Sianturi R.S.G., Wina E., Kusumaningrum D.A. (2016). Chitosan nanoparticle of hCG (Human Chorionic Gonadotrophin) hormone in increasing induction of dairy cattle ovulation. JITV.

[45] Gonzalez-Bulnes A., Astiz S., Parraguez V.H., García-Contreras C., Vazquez-Gomez M. (2016). Empowering translational research in fetal growth restriction: Sheep and swine animal models. Curr. Pharm. Biotechnol..

[46] Gonzalez-Bulnes A., Astiz S., Ovilo C., Lopez-Bote C.J., Torres-Rovira L., Barbero A., Ayuso M., Garcia-Contreras C., Vazquez-Gomez M. (2016). Developmental origins of health and disease in swine: Implications for animal production and biomedical research. Theriogenology.

[47] Lopez-Tello J., Arias-Alvarez M., Gonzalez-Bulnes A., Sferuzzi-Perri A.N. (2019). Models of Intrauterine growth restriction and fetal programming in rabbits. Mol. Reprod. Dev..

[48] Scifres C.M., Nelson D.M. (2009). Intrauterine growth restriction, human placental development and trophoblast cell death. J. Physiol..

[49] Zhang B., Liang R., Zheng M., Cai L., Fan X. (2019). Surface-functionalized nanoparticles as efficient tools in targeted therapy of pregnancy complications. Int. J. Mol. Sci..

[50] de Araújo T.E., Milián I.C.B., de Souza G., da Silva R.J., Rosini A.M., Guirelli P.M., Franco P.S., Barbosa B.F., Ferro E.A.V., da Costa I.N. (2020). Experimental models of maternal-fetal interface and their potential use for nanotechnology applications. Cell Biol. Int..

[51] Valero L., Alhareth K., Gil S., Lecarpentier E., Tsatsaris V., Mignet N., Fournier T., Andrieux K. (2018). Nanomedicine as a potential approach to empower the new strategies for the treatment of preeclampsia. Drug Discov. Today.

[52] Refuerzo J.S., Longo M., Godin B. (2017). Targeted nanoparticles in pregnancy: A new frontier in perinatal therapeutics. Am. J. Obstet. Gynecol..

[53] Paul J.W., Hua S., Ilicic M., Tolosa J.M., Butler T., Robertson S., Smith R. (2017). Drug delivery to the human and mouse uterus using immunoliposomes targeted to the oxytocin receptor. Am. J. Obstet. Gynecol..

[54] Ray P.C., Yu H., Fu P.P. (2009). Toxicity and environmental risks of nanomaterials: Challenges and future needs. J. Environ. Sci. Health.

